# The association between social care expenditure and multiple-long term conditions: A population-based area-level analysis

**DOI:** 10.1177/26335565231208994

**Published:** 2023-10-27

**Authors:** Emeka Chukwusa, Paulino Font-Gilabert, Jill Manthorpe, Andrew Healey

**Affiliations:** 1 Cicely Saunders Institute, 4616 King’s College London, London, UK; 2 Health Services and Population Research Department, Institute of Psychiatry, Psychology & Neuroscience, The David Goldberg Centre, 4616 King’s College London, London, UK; 3 NIHR Policy Research Unit in Health and Social Care Workforce, 4616 King’s College London, London, UK

**Keywords:** Prevalence, older people, multiple long-term conditions, social care expenditure, social care, local authority, death certificate

## Abstract

**Background:**

Multiple long-term health conditions (MLTCs) are common and increasing among older people, yet there is limited understanding of their prevalence and association with social care expenditure.

**Aim:**

To estimate the prevalence of MTLCs and association with English social care expenditure.

**Methods:**

Our study population included those aged ≥ 65 who died in England in the year 2018 with any of the following long-term conditions recorded on their death certificate: diabetes; cardiovascular diseases (CVDs) including hypertension; dementia; stroke; respiratory; and chronic kidney diseases (CKDs). Prevalence was based on the proportion of death reported for older people with MTLCs (≥ 2) in each of the 152 English Local Authorities (LAs). Ordinary least square regression (OLS) was used to assess the relationship between prevalence of MTLCs and adult social care expenditure, adjusting for LA characteristics.

**Results:**

Of the 409551 deaths reported, 19.9% (n = 81395) had ≥ 2 MTLCs, of which the combination of CVDs-diabetes was the most prevalent. Hospitals were the leading place of death for those with MTLCs. Results from the OLS regression model showed that an increased prevalence of MLTCs is associated with higher LA social care expenditure. A percentage point increase in prevalence of MLTCs is associated with an increase of about £8.13 in per capita LA social care expenditure.

**Conclusion:**

Our findings suggest that the increased prevalence of MTLCs is associated with increased LA social care expenditure. It is important for future studies to further explore the mechanisms or link between LA social care expenditure and the prevalence of MTLCs.

## Background

Multimorbidity, or the co-occurrence of Multiple Long-Term Conditions (MTLCs), is highly prevalent among older people.^1^ An estimated one-third of adults living in community settings have MTLCs.^2^ In England, UK, with population ageing, the percentage of people living with MTLCs is estimated to rise to 17.0% by 2035 (from a prevalence of 9.8% in 2005).^3^ This projected increase has implications for the provision of and demand for publicly funded health and social care services. Moreover, increased demand for health and care systems has cost implications.^4^ For example, a systematic review showed that having more MTLCs is associated with higher costs.^5^

While several studies have explored determinants of MTLCs and overall health care costs,^1,6^ less is known about the association between prevalence of MLTCs and publicly funded social care expenditure. To date, most studies have focused on the health care costs of various disease combinations. For example, Hajat and colleagues explored the costs of common disease clusters and concluded that clusters including HIV/AIDS, mental and behavioural disorders, renal failure, and CVDs are the main components of US health care spending.^7^ Doos and colleagues showed that chronic heart failure and COPD are associated with high costs, and hospital admissions, compared with the cost of index conditions.^8^

In terms of social care costs, one ecological study conducted in England, UK, investigated area-level expenditure in the context of public spending cuts and discovered that a 1% reduction in per capita total social service expenditure in a Local Authority (LA) was related to a 0.1% rise in the prevalence of multimorbidity.^9^ A recent study using individual-level social care expenditure data for Somerset, England, explored whether multimorbidity observed in the same individual would cost the social care system more or less than the additive cost of two individuals with singular conditions.^10^ They found no evidence to support this; however, it is unclear whether this can be extrapolated more widely.

Social care can play a crucial role in the wellbeing of older people due to the often long-term nature of their illnesses and care needs.^11^ In England, a request for LA-funded social care must go through a needs assessment and a means test, and only requests for those individuals with the greatest requirements and financial assets below national thresholds are likely to receive assistance^12^ According to a recent King’s Fund report, most recipients of LA-funded social care are aged 65 and over, with physical support constituting the largest need for care, while people with learning disabilities are the major group of recipients of LA-funded care aged 18-64 years.^12^

Social care differs from National Health Service (NHS) care, which is non-means tested and centrally funded, in its responsibility for activities such as aiding with activities of daily living and promoting wellbeing among people with care and support needs. Many people with care and support need to pay for their own care privately in England, while others receive publicly funded or LA-funded social care from for-profit companies. Over the years, demand for LA social care support has risen, but gross expenditure remains low.^13^ This means it may be helpful to uncover any link between prevalence of MTLCs and LA social care expenditure. This may shed light on any mismatch or variations between social care expenditure and services and care needs among people with MTLCs.

In this study, we aimed to understand the association and prevalence of MLTCs, describe the sociodemographic and clinical characteristics of individuals with MTLCs, including place of death, and estimate a simple model to examine the relationship between MLTCs and level of LA expenditure on the provision of social care services for their older populations who are eligible for LA-funded support. We hypothesised that LAs with populations with a greater prevalence of MLTCs are likely to incur more social care expenditure. This would reinforce the importance of aligning resources to health and care needs.

## Methods

### Study design and settings

A population-based area-level analysis in England, UK. The relationship between social care expenditure and prevalence of MTLCs was analysed at LA-level. In England, there are 152 LAs or Councils with Adult Social Services Responsibilities (CASSRs). We used the LA as the unit of analysis because adult social care expenditure and provision are reported at aggregate LA level in England.^14^ The study followed the STROBE Statement for reporting observational studies using routinely collected health data.^15^

### Data source and study population

The dataset comprised records of the deaths of older people aged 65 years and above in England in 2018. We focused on those aged 65 years and above, as this is the age group at greatest risk of developing MTLCs.^16,17^

The dataset was extracted from the Office for National Statistics (ONS) death registry. The dataset included information about the deceased recorded at the time of death. It comprises gender (male and female), marital status (divorced, married, separated/dissolved, single, unknown/not stated, and widowed), place of settlement at the time of death (urban & rural), government office region of residence (East, East-Midlands, London, North-east, North-west, South-east, South-west, West Midlands, and Yorkshire and the Humber), underlying & contributory causes of death, place of death (care homes, hospitals, elsewhere, and home), and socioeconomic status (SES) derived from index of multiple deprivation (IMD) scores. The IMD is an area-based estimate of socioeconomic status for each Lower Super Output Area (LSOA). LSOAs are geographical areas with a population of roughly 1500 people each. The IMD scores were grouped into quintiles, ranging from most deprived (1) to least deprived (5), for descriptive analysis to enhance the intelligibility of the results. The underlying and contributory cause of death was recorded according to the International Statistical Classification of Diseases, Injuries, and Causes of Death (ICD) 10th edition (ICD-10),. Underlying causes of death include cancers (C00 - C97), cerebrovascular diseases (CBDs: G45–G46, I60–I69); Chronic Obstructive Pulmonary Diseases (COPD: J40-J44, J47); cardiovascular diseases (CVDs: I00-I52, I70-I99); neurological conditions (G35-G37, G20, F02.3, G12). The reported contributing causes of death recorded on the death certificate were used as a proxy for MTLCs.^17,18^ We identified six MTLCs used in a previous study,^18^ according to their ICD-10 codes, including: chronic respiratory disease (J4, J6, J7, J82, and J84); diabetes (E10 – E14); cardiovascular disease, including hypertension (CVDs: I1, I20, I25, I7, and I8); dementia (F00, F01, F02, F03, G30 – G32 and R54); stroke and other cerebrovascular conditions (I6x); and chronic kidney disease (CKDs: N11, N18, and N19).

**Figure 1.**
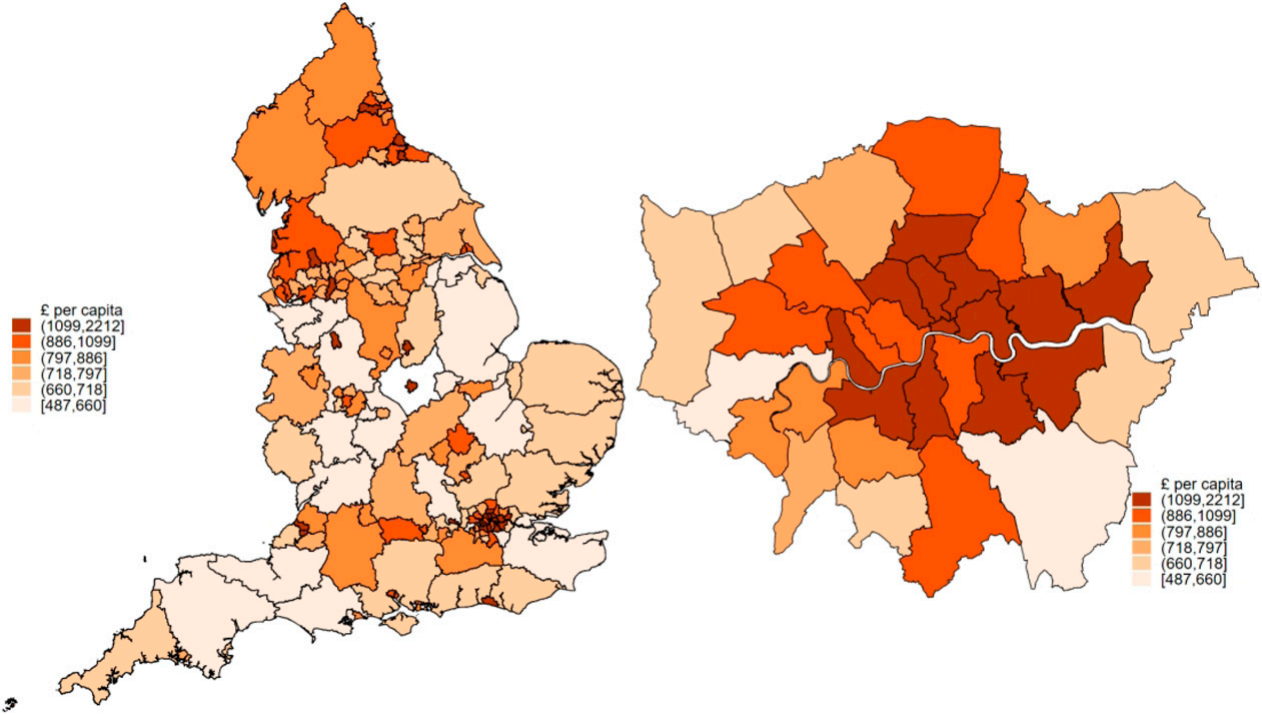
Geographic variations in social care expenditure per capita (£) in England, UK. Social care expenditure across local authorities in England UK for the period: 2018/19. Local authority boundaries are the English Upper-Tier Local Authorities 2016 version. The denominator comprised Population aged 65 and over in each local authority area. The map on the right show variations across local authorities in London, UK.

Social care expenditure data (Figure 1) were obtained from NHS Digital’s Adult Social Care Activity and Finance Report for the period of 2018-19.^19^ Adult social care expenditure is defined as expenditure “on sickness and disability and personal social service expenditure on old age.”^20^ The data comprised aggregate expenditures for the population aged 65 and above.^21^ Our analysis used gross current expenditures, which comprised the amount spent by LAs that is not offset by income from clients (many of them pay on a means-tested basis) and excluding capital charges (i.e., the cost of the buildings and equipment needed to deliver services).

We derived the median LA-level age and the share of female gender among individuals aged 65 and above from the ONS mid-2018 population estimates data.^22^ The ONS mid-2018 population data comprised population estimates for each LA by age and sex. LA income was calculated from the Department of Housing, Communities, and Local Government revenue outturn (RO) 2018-19 service expenditure summary data (now the Department for Levelling up, Housing, and Communities).^23^ The data comprise sales, fees, and charges, as well as recharges and funding transfers from other central government and delivery bodies, such as the NHS Better Care Fund.^23^ LA deprivation data were calculated from the 2019 English IMD scores. For regression modelling, we derived mean deprivation scores for each LA by averaging the IMD scores of LSOAs within LAs.

### Study variables

The *per capita* social care expenditure of adults aged 65 and over in each LA was the dependent variable. The prevalence of MTLCs was the main explanatory variable, controlling for age, gender, share of females, LA incomes, and IMD scores. We derived prevalence by calculating the proportions of decedents aged 65 and older with ≥ 2 MTLCs aggregated across all 152 English LAs. The selection of explanatory variables was guided by determinants of social care expenditure used in previous studies.^10,14^ The rationale for controlling for LA income arises from the fact that higher resources allow for higher spending on social care (irrespective of the demand or need for social care services from the local population). That is, LA income is a proxy for the capacity of LAs to supply social care services. Similarly, we included deprivation scores to capture much of the local environment that might affect both the demand and ability to supply care by LAs (including infrastructure and capacity to provide services, overall population health, and a measure of the income level of residents, among others). We included regions to account for sociopolitical factors such as regional-level policies that might influence how much resources LAs devote to social care expenditures. Finally, controls for differences across LAs in the sex composition of the population and the median age are needed to account for other sex- or age-induced differences in the need for social care. Age, gender, and deprivation are important predictors of social care expenditures.^10,24^

### Statistical analysis

Sociodemographic and clinical characteristics of our study population were described using mean and standard deviations (SD) for continuous variables and counts and percentages for categorical variables. The proportions of the study population with or without MTLCs were stratified by counts of zero, one, or ≥ 2.

We used ordinary least squares regression (OLS) to quantify the association between the prevalence of MTLCs and social care expenditure at the LA level using equations 1- 5. Equation (1) is the baseline equation and provides the unconditional association between the prevalence of MTLCs and social care expenditures in the LA. Equations 2-5 show the successive inclusion of LA per capita income, deprivation scores, government office regions, LA median age, and the share of females in the population aged 65 and above.

(1) $$E_{i}=\alpha_{1}+\beta_{1}{MLTC}_{i}+\varepsilon_{i}$$

Where $E_{i}$ is the per capita social care expenditure of LA i on individuals aged 65 and above in the period of 2018-19, and $\varepsilon_{i}$ is the error term. We also estimate different versions of the baseline model that include covariates:

(2) $$E_{i}=\alpha_{2}+{\beta_{2}MLTC}_{i}+{{µ}_{2}I}_{i}+\varepsilon_{i}$$

(3) $$E_{i}=\alpha_{3}+{\beta_{3}MLTC}_{i}+{{{µ}_{3}I}_{i}+{\gamma_{3}D}_{i}+\varepsilon }_{i}$$

(4) $$E_{i}=\alpha_{4}+{\beta_{4}MLTC}_{i}+{{µ}_{4}I}_{i}+{\gamma_{4}D}_{i}+\overset{9}{\underset{j=1}{\sum }}{\Omega_{4}R}_{j}+\varepsilon_{i}$$

(5) $$E_{i}=\alpha_{5}+{\beta_{5}MLTC}_{i}+{µ}_{5}I_{i}+{\gamma_{5}D}_{i}+\overset{9}{\underset{j=1}{\sum }}{\Omega_{5}R}_{j}+{\sigma_{5}S}_{i}+{\tau_{5}A}_{i}+\varepsilon_{i}$$

Where $I_{i}$ is the income per capita of LA i , $D_{i}$ is the deprivation score, $R_{j}$ are region dummies (formerly known as Government Office regions, the highest tier of sub-national division in England), $S_{i}$ is the share of females, and $A_{i}$ is the median age of the population aged 65 and above. Confidence intervals are reported at the 95% confidence level. We estimated robust standard errors to address heteroskedasticity in the variance of the residual (p-value of 0.057 in the Breusch–Pagan/Cook–Weisberg test for heteroskedasticity). We conducted a robustness check in our model by changing the order in which covariates were adjusted for in the model, i.e., introducing LA income, IMD average score, share of females, median age, and regional effects before prevalence of MLTCs.

All descriptive analyses, including the derivation of MTLC clusters, were completed using R version 4.1.2.^25^ OLS regression was carried out in Stata/IC 17 (STATA, College Station, TX).

### Ethical approval and permission

The study used fully anonymised datasets and did not involve direct individual contact. Hence, consent to participate was not required. In addition, Emeka Chukwusa (EC) is accredited to analyse this data through the ONS Approved Researcher Scheme.

### Patient and public involvement

A draft of this paper was reviewed by a public and patient involvement (PPI) representative who has interest in national services for older people. She made suggestions for some areas to improve clarity which have been adopted. The projects undertaken by the study team under this funding programme have input from a PPI panel.

## Results

### Sociodemographic and clinical characteristics of study population

Table 1 summarises the sociodemographic and clinical characteristics of the study population. There were 409,551 people aged ≥ 65 years with chronic respiratory diseases, diabetes, CVDs, dementia, stroke, and CKDs as secondary causes of deaths recorded on their death certificates in 2018 (see Table 1). More than half of them had at least one MTLC, and almost a fifth had at least ≥ 2 MTLCs (n = 81,395, 19.9% of 409,551). Most were female, but slightly more people with ≥ 2 MLTCs were male (n = 41,363, 50.8% of 81,395). The mean age of those with ≥ 2 MTLCs was 84.03 years (SD: 8.05). The proportion of people with ≥2 MTLC increased with age, with those aged ≥ 85 years accounting for more than half (n = 42,249, 51.9% of 81,395). Other than deaths from other causes, cancer (n =10,399, 12.8% of 81,395) and CVDs (n = 25,328, 31.1% of 81,395) were the two main underlying causes of death for those with ≥ 2MTLCs. Most of the deaths recorded of people with ≥ 2 MTLCs lived in the Southeast region (n = 13,193, 16.2% of 81,395).

**Table 1.** Characteristics of study cohort by number of Multiple Long-term conditions in England 2018 (n = 409551).

Variables	level	Overall	MTLCs		
			0	1	2+
N		409551	174186 (42.5 )†	153970 (37.6)†	81395 (19.9)†
Gender (%)	Female	216120 (52.8)	92731 (53.2)	83357 (54.1)	40032 (49.2)
	Male	193431 (47.2)	81455 (46.8)	70613 (45.9)	41363 (50.8)
Age in years (mean (SD))		83.2 (8.65)	81.5 (8.77)	84.5 (8.52)	84.0 (8.05)
Age group (%)	65-74	78927 (19.3)	43696 (25.1)	23395 (15.2)	11836 (14.5)
	75-84	135002 (33.0)	61067 (35.1)	46625 (30.3)	27310 (33.6)
	85+	195622 (47.8)	69423 (39.9)	83950 (54.5)	42249 (51.9)
Marital status (%)	Divorced	40368 (9.9)	18104 (10.4)	14346 (9.3)	7918 (9.7)
	Married	151802 (37.1)	71576 (41.1)	51414 (33.4)	28812 (35.4)
	Separated/Dissolved	486 (0.1)	234 (0.1)	182 (0.1)	70 (0.1)
	Single	27911 (6.8)	12287 (7.1)	10713 (7.0)	4911 (6.0)
	Unknown/Not stated	2242 (0.5)	982 (0.6)	828 (0.5)	432 (0.5)
	Widowed	186742 (45.6)	71003 (40.8)	76487 (49.7)	39252 (48.2)
Cause of death (%)	Cancers	106549 (26.0)	71335 (41.0)	24815 (16.1)	10399 (12.8)
	*CBDs	25794 (6.3)	8856 (5.1)	11208 (7.3)	5730 (7.0)
	*COPDs	24958 (6.1)	5477 (3.1)	10645 (6.9)	8836 (10.9)
	*CVDs	77373 (18.9)	14874 (8.5)	37171 (24.1)	25328 (31.1)
	Neurological conditions	8049 (2.0)	3521 (2.0)	3479 (2.3)	1049 (1.3)
	Other causes of deaths	166828 (40.7)	70123 (40.3)	66652 (43.3)	30053 (36.9)
Place of death (%)	Care Homes	108783 (26.6)	44882 (25.8)	45750 (29.7)	18151 (22.3)
	Homes	89780 (21.9)	41520 (23.8)	32922 (21.4)	15338 (18.8)
	Hospices	20896 (5.1)	15740 (9.0)	3545 (2.3)	1611 (2.0)
	Hospital	185881 (45.4)	70046 (40.2)	70152 (45.6)	45683 (56.1)
	Others	4211 (1.0)	1998 (1.1)	1601 (1.0)	612 (0.8)
Settlement (%)	Rural	86287 (21.1)	38165 (21.9)	32401 (21.0)	15721 (19.3)
	Urban	323264 (78.9)	136021 (78.1)	121569 (79.0)	65674 (80.7)
Socioeconomic status (%)	1 (Most deprived)	73803 (18.0)	29541 (17.0)	27612 (17.9)	16650 (20.5)
	2	78713 (19.2)	32197 (18.5)	29508 (19.2)	17008 (20.9)
	3	86566 (21.1)	36800 (21.1)	32738 (21.3)	17028 (20.9)
	4	86198 (21.0)	37458 (21.5)	32716 (21.2)	16024 (19.7)
	5 (Least Deprived)	84271 (20.6)	38190 (21.9)	31396 (20.4)	14685 (18.0)
Regions (%)	East	48548 (11.9)	19652 (11.3)	18800 (12.2)	10096 (12.4)
	East-Midlands	37388 (9.1)	15780 (9.1)	13607 (8.8)	8001 (9.8)
	London	38453 (9.4)	15748 (9.0)	13938 (9.1)	8767 (10.8)
	North-East	22793 (5.6)	8512 (4.9)	9586 (6.2)	4695 (5.8)
	North-West	57544 (14.1)	27166 (15.6)	20300 (13.2)	10078 (12.4)
	South-East	68291 (16.7)	29290 (16.8)	25808 (16.8)	13193 (16.2)
	South-West	48081 (11.7)	21046 (12.1)	19287 (12.5)	7748 (9.5)
	West midlands	45630 (11.1)	18063 (10.4)	16888 (11.0)	10679 (13.1)
	Yorkshire and The Humber	42823 (10.5)	18929 (10.9)	15756 (10.2)	8138 (10.0)

* *CBDs: Cerebrovascular diseases; COPD: Chronic Obstructive Pulmonary Diseases; CVDs: Cardiovascular Diseases*.

† The values are based on row percentages.

### Prevalence of multiple long-term conditions (≥ 2 conditions)

**Figure 2.**
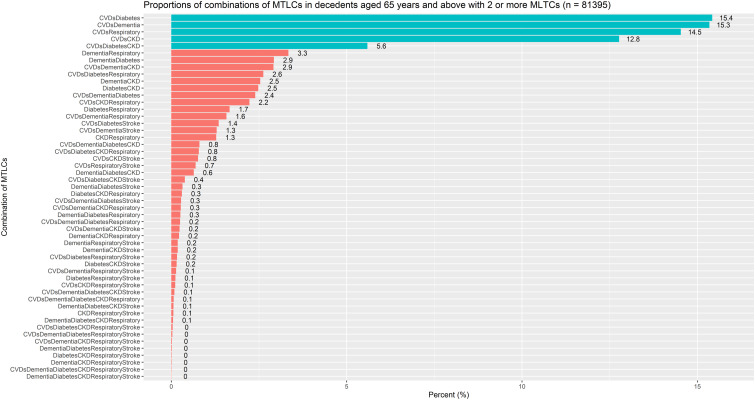
Proportions of combinations of MTLCs in decedents aged 65 years and above with 2 or more MTLCs (n = 81395).

We identified a total of 52 unique MTLC combinations (Figure 2, Table S1) in our study population (n = 81,395). The combination of CVDs-diabetes (15.4%) was the most prevalent, followed closely by CVDs-dementia (15.3%), CVDs-respiratory diseases (14.5%) and CVDs-CKDs (12.8%).

Table 2 presents the sociodemographic characteristics of the study population stratified by the five most common combinations of MTLCs (n = 51,817). The mean age at death was 83.6 years (SD ±8.18). Slightly over half were men (n = 26,977, 52.1% of 51,817). MTLCs, comprising CVDs and dementia, were common among female decedents (n = 7,767, 62.2 of 12,489). The prevalence of conditions comprising CVDs-diabetes-CKDs (22.5% to 15.9%) or CVDs - respiratory diseases (24.4% to 15.8%) decreased by socioeconomic quintile, from the most to the least deprived quintile.

**Table 2.** Characteristics of study cohort by top 5 combinations of MLTCs (n = 51817).

Variables	Level	Overall	*CVDs - CKD	CVDs - Dementia	CVDs – Diabetes	CVDs - Diabetes - CKD	CVDs - Respiratory
N (%)		51817	10394 (20.1)†	12489 (24.1)†	12555 (24.2)†	4553 (8.9)†	11826 (22.8)†
Gender (%)	Female	24840 (47.9)	4888 (47.0)	7767 (62.2)	5339 (42.5)	1929 (42.4)	4917 (41.6)
	Male	26977 (52.1)	5506 (53.0)	4722 (37.8)	7216 (57.5)	2624 (57.6)	6909 (58.4)
Age in years (mean (SD))		83.57 (8.18)	85.06 (7.44)	89.30 (6.00)	80.63 (7.87)	81.30 (7.57)	80.20 (7.69)
Age group (%)	65-74	8416 (16.2)	1057 (10.2)	220 (1.8)	3139 (25.0)	947 (20.8)	3053 (25.8)
	75-84	17680 (34.1)	3336 (32.1)	2228 (17.8)	5129 (40.9)	1933 (42.5)	5054 (42.7)
	85+	25721 (49.6)	6001 (57.7)	10041 (80.4)	4287 (34.1)	1673 (36.7)	3719 (31.4)
Marital status (%)	Divorced	5081 (9.8)	799 (7.7)	753 (6.0)	1377 (11.0)	451 (9.9)	1701 (14.4)
	Married	19012 (36.7)	3835 (36.9)	3146 (25.2)	5310 (42.3)	1970 (43.3)	4751 (40.2)
	Separated/Dissolved	45 (0.1)	13 (0.1)	< 10*	< 10*	< 10*	13 (0.1)
	Single	3261 (6.3)	607 (5.8)	698 (5.6)	897 (7.1)	297 (6.5)	762 (6.4)
	Unknown/Not Stated	289 (0.6)	38 (0.4)	< 10*	< 10*	< 10*	105 (0.9)
	Widowed	24129 (46.6)	5102 (49.1)	7844 (62.8)	4873 (38.8)	1816 (39.9)	4494 (38.0)
Underlying cause of death (%)	Cancers	6680 (12.9)	1209 (11.6)	930 (7.4)	2112 (16.8)	427 (9.4)	2002 (16.9)
	CBDs	3735 (7.2)	472 (4.5)	1482 (11.9)	1136 (9.0)	194 (4.3)	451 (3.8)
	COPDs	4678 (9.0)	287 (2.8)	531 (4.3)	387 (3.1)	77 (1.7)	3396 (28.7)
	CVDs	17167 (33.1)	3988 (38.4)	3921 (31.4)	4222 (33.6)	1531 (33.6)	3505 (29.6)
	Neurological conditions	530 (1.0)	37 (0.4)	342 (2.7)	88 (0.7)	10 (0.2)	53 (0.4)
	Other causes of deaths	19027 (36.7)	4401 (42.3)	5283 (42.3)	4610 (36.7)	2314 (50.8)	2419 (20.5)
Place of death (%)	Care Homes	9710 (18.7)	1151 (11.1)	5147 (41.2)	2020 (16.1)	427 (9.4)	965 (8.2)
	Homes	10401 (20.1)	1500 (14.4)	2192 (17.6)	2973 (23.7)	783 (17.2)	2953 (25.0)
	Hospices	1087 (2.1)	328 (3.2)	89 (0.7)	255 (2.0)	144 (3.2)	271 (2.3)
	Hospital	30194 (58.3)	7360 (70.8)	4988 (39.9)	7160 (57.0)	3174 (69.7)	7512 (63.5)
	Others	425 (0.8)	55 (0.5)	73 (0.6)	147 (1.2)	25 (0.5)	125 (1.1)
Settlement (%)	Rural	10090 (19.5)	2093 (20.1)	2722 (21.8)	2443 (19.5)	763 (16.8)	2069 (17.5)
	Urban	41727 (80.5)	8301 (79.9)	9767 (78.2)	10112 (80.5)	3790 (83.2)	9757 (82.5)
Socioeconomic status (%)	1 (Most deprived)	10299 (19.9)	1796 (17.3)	1923 (15.4)	2666 (21.2)	1024 (22.5)	2890 (24.4)
	2	10746 (20.7)	2106 (20.3)	2387 (19.1)	2678 (21.3)	1004 (22.1)	2571 (21.7)
	3	10939 (21.1)	2300 (22.1)	2696 (21.6)	2629 (20.9)	941 (20.7)	2373 (20.1)
	4	10281 (19.8)	2137 (20.6)	2736 (21.9)	2429 (19.3)	860 (18.9)	2119 (17.9)
	5 (Least Deprived)	9552 (18.4)	2055 (19.8)	2747 (22.0)	2153 (17.1)	724 (15.9)	1873 (15.8)
Regions (%)	East	6497 (12.5)	1201 (11.6)	1711 (13.7)	1605 (12.8)	553 (12.1)	1427 (12.1)
	East-Midlands	5050 (9.7)	1119 (10.8)	1159 (9.3)	1286 (10.2)	428 (9.4)	1058 (8.9)
	London	5742 (11.1)	1131 (10.9)	1189 (9.5)	1566 (12.5)	657 (14.4)	1199 (10.1)
	North-East	2783 (5.4)	438 (4.2)	756 (6.1)	650 (5.2)	193 (4.2)	746 (6.3)
	North-West	6548 (12.6)	1368 (13.2)	1197 (9.6)	1537 (12.2)	563 (12.4)	1883 (15.9)
	South-East	8534 (16.5)	1778 (17.1)	2150 (17.2)	2009 (16.0)	699 (15.4)	1898 (16.0)
	South-West	4809 (9.3)	848 (8.2)	1517 (12.1)	1083 (8.6)	362 (8.0)	999 (8.4)
	West midlands	6619 (12.8)	1341 (12.9)	1642 (13.1)	1573 (12.5)	646 (14.2)	1417 (12.0)
	Yorkshire- the Humber	5235 (10.1)	1170 (11.3)	1168 (9.4)	1246 (9.9)	452 (9.9)	1199 (10.1)

* *Cardiovascular diseases (CVDs) and chronic kidney diseases (CKDs)*.

† The values are based on row percentages. *These values are suppressed to avoid secondary disclosure.

Hospitals were the leading place of death for older people, with the top five most common combinations of MTLCs (n = 30,194, 58.3% of 51,817), followed by deaths at home (n = 10401, 20.1% of 51817) and deaths in care homes (n = 9,710, 18.7% of 51, 817).Those with a combination of CVDs-dementia, died mainly in care homes.

### Association between prevalence of MTLCs  and social care expenditure in England, UK

Table 3 shows the results of OLS regression of *per capita* social care expenditure on the prevalence of MLTCs at the LA level. Column 1 shows that a 1 percentage point (pp) increase in the prevalence of MLTC is associated with an increase of about £17.4 in *per capita* social care expenditure (statistically significant at the 1% level). The association between MTLCs and social care expenditure persists even after controlling for characteristics of the LA, including income *per capita* (Column 2) and the deprivation score (Column 3). Column 4 includes region fixed effects. Column 5 further includes the share of females and the median age of the population aged 65 and above. Across regions in England, a 1 pp increase in the prevalence of MTLCs is associated with a £8.13 increase in social care expenditure *per capita* (statistically significant at the 5% level). Changing the order in which the explanatory variables were accounted for in the model did not affect the magnitude or direction of the association between the prevalence of MTLCs and social care expenditure (Table S2).

**Table 3.** Association between MTLCs and LA social care expenditure.

Social care expenditure					
VARIABLES	(1)	(2)	(3)	(4)	(5)
MLTC pp	17.37*** (7.67 - 27.06)	17.22*** (7.69 - 26.75)	13.16*** (4.06 - 22.27)	8.67** (1.00 - 16.33)	8.13** (0.24 - 16.02)
LA income pc		0.04* (−0.00 - 0.08)	0.04** (0.00 - 0.07)	0.03* (−0.00 - 0.06)	0.01 (−0.02 - 0.05)
IMD - Average score			10.91*** (6.87 - 14.95)	15.36*** (10.34 - 20.38)	15.86*** (10.82 - 20.90)
Share female (pp)					−36.00 (−119.95 - 47.94)
Median age (65+)					−4.78 (−79.76 - 70.20)
Constant	498.11***	478.71***	312.02***	319.76***	2,634.47
	(302.53 - 693.68)	(289.03 - 668.39)	(89.01 - 535.03)	(128.55 - 510.96)	(−1,805.59 - 7,074.53)
Observations	152	151	148	148	148
R-squared	0.08	0.12	0.24	0.39	0.40
Region FE				YES	YES

Results from OLS regressions. MLTC pp is the prevalence of multiple long-term conditions expressed in percentage points. LA income pc is the local authority income per capita. Deprivation score is the index of multiple deprivation, share female (pp) is the proportion of female population. Robust confidence Interval in parentheses ***p < 0.01, **p < 0.05, *p < 0.1.

## Discussion

### Main findings

In this population-based area-level analysis, we found a positive association between the prevalence of MTLCs and aggregate-level LA social care expenditure even after controlling for LA income *per capita*, deprivation scores, gender, and median LA population age. This finding suggests that social care expenditure is unsurprisingly linked with the prevalence of MTLCs since English LA social care expenditure has a threshold of eligibility related to population need under the Care Act 2014. Caution must be exercised in interpreting this finding as our regression model did not account for several potentially influential covariates such as the frequency of care interventions (the type of care provided could range from some home care support, day service attendance, or round-the-clock residential or nursing home care) or the proportion of older people eligible for LA funding in each LA. In terms of prevalence, our study showed that the combination of cardiovascular disease and diabetes (CVDs-diabetes) was the most prevalent condition in our study population. We also found that cardiovascular diseases co-occurred mostly with dementia, CKD, and respiratory diseases. A recent systematic review and meta-analysis, including 59 studies from high-income countries, concluded that disease combinations comprising diabetes and heart/vascular conditions are the most costly for healthcare systems.^11^

We found a decrease in the prevalence of MTLCs (i.e., comprising CVDs, diabetes, and CKDs or CVDs and respiratory diseases) by level of socioeconomic status, with a higher prevalence of these conditions concentrated in the poorest areas. This result is consistent with previous studies showing that the prevalence of multimorbidity is higher in deprived localities.^26–28^ One possible reason for the increased prevalence of clusters of MTLCs in deprived areas may be the relative concentration of common stressors, inequalities, and intermediate factors such as environmental exposures.^29,30^

In terms of place of death, hospitals were the main place of death for older people with and without MLTCs. This finding corroborates a study that showed that the recording of a secondary cause of death – a proxy for MTLCs - was more frequent in hospitals.^31^ The place of death may be a quality indicator for the care received at the end-of-life. However, dying at home or out-of-hospital is considered preferable by many older people. Achieving this preference is difficult for those with MTLCs, who often experience multiple unplanned admissions at the end-of-life^32^ due to unpredictable disease trajectories. Hospitals being the most common place of death for older people with MTLCs is not surprising. However, it remains unclear whether people with MTLCs who died in hospitals might be better cared for in the community. For example, there is evidence that access to community-based specialist palliative care reduces hospital deaths at the end-of-life.^33^ Therefore, having good access to social care, including palliative care,^34,35^ better understanding of the complex care needs of people, and early communication of end-of-life care preferences between individuals, families, and health or social care professionals could increase the chances of dying at home or out of hospital, where wanted.^36^

### Comparison with other studies

Research exploring the links between MTLCs, and care costs has focused mainly on overall health costs.^1,6^ Only a few studies have explored the association between MTLCs and social care expenditure,^10,37,38^ some of which used limited samples in specific LAs located in southern England. For example, from the Symphony project, Kasterids and colleagues showed that in South Somerset (England) multimorbidity (and not age) was the key driver of social care (and health care) costs.^37^ Similarly, examining social care costs alone in the same region, Blawat and colleagues found the social care costs for a person with two co-morbidities were not statistically different than those for people with just one chronic condition.^10^ Using survey data for England, and although not directly inspecting the association between MLTCs and social care costs, Nikolova and colleagues found a non-linear relationship between social care costs and frailty.^38^ That is, as might be expected, social care costs increase more rapidly when a person has higher levels of frailty. Our finding of an increased prevalence of MTLCs with social care expenditure supports the findings of previous studies. Our data comprise a nationally representative population of decedents and social care expenditures for all LAs in England with adult social care responsibilities. In addition, our data showed that the association between social care expenditure and the prevalence of MTLCs is generalisable to other LAs in England. Although, there is likely to be heterogeneity in the association across LAs,^39^ this could be partly due to differences in the ‘spending power’ of LAs.^40^ In England, while there is little centralised funding for adult social care; some LA expenditure is financed through its own generated revenue.^41^ This suggests that LAs that are more affluent with more spending power and fewer social care needs are likely to fare better than their less affluent counterparts. This could exacerbate inequalities across regions and further aggravate the North-South divide in health outcomes in England. For instance, the prevalence of MLTCs has been increasing more notably in Northern regions.^28^

### Implication of findings

The results show that the existence and evolution of MTLCs, including social care needs, should be considered at the time of allocating social care funds. The regression estimates also suggest that national care responsibilities (devolved as they are to LA level) might in effect discriminate against people based on geographical location,. That is, LAs with more spending power might provide more social care services, and a lack of resources might prevent other LAs from increasing people’s care packages.

The positive relationship between the prevalence of MTLCs and social care expenditure suggests that any mismatch between expenditure and social care needs caused by fiscal cuts or reductions in LA income may increase the likelihood of older people with MTLCs having unmet social needs. Several studies using empirical modelling data have shown that reduction in spending exacerbates needs both in health and social care.^9,42^ Stoke and colleagues showed that a reduction in *per capita* total service expenditure by 1% was associated with a 0·10% increase in the prevalence of multimorbidity.^9^ In another study, Watkins and colleagues found that fiscal constraints seemed to lead to higher-than-expected number of deaths, and especially increased numbers of deaths in care homes and at home.^42^

We found that cardiovascular diseases co-occurred with other long-term conditions. This finding has implications for services’ organisation and delivery. Most healthcare systems are designed to treat a single disease - a model that fails most older people with MTLCs.^18,43^ Models of care that involve service integration or close working collaborations with health professionals working in cardiology, diabetes, and renal support services may optimise care for people with MTLCs.

We found that the prevalence of certain disease combinations (CVDs-diabetes-CKDs and CVDs- respiratory diseases) increased by socioeconomic quintile, suggesting that older people with these long-term conditions are at risk of social economic disadvantage. This finding highlights the need for more care integration in deprived areas for people with MTLCs. Policy reform initiatives and interventions targeted at deprived areas have the potential to improve the quality of care received by people with MTLCs,^44^ although this would seem unlikely to be achievable by LAs alone. There is evidence that care models such as the CARE-PLUS, comprising a “whole-system approach”^45^ including structured longer primary care appointments, care continuity, practitioner support, and self-management patient care, can improve care outcomes for people with MTLCs living in deprived areas. For example, the CARE-PLUS care model, implemented in very deprived areas of Glasgow, Scotland, has been shown to improve the quality of care.^44^

### Strengths and limitations

This population-based area-level analysis was based on a nationally representative sample of all deaths registered in England, UK, in 2018. This means that our study findings can be generalised across the UK population. A major strength of this study is the use of patient-level datasets aggregated to the LA level, which is the spatial unit for the provision of means-tested and eligibility-tested social care in England.^46^ In addition, our dataset was extracted before the start of the COVID-19 pandemic, meaning that the prevalence of MTLCs was not influenced by excess deaths associated with the pandemic.^47^

Our study has several limitations. First, our study design is observational; thus, we cannot infer any causality between the observed association of LA social care expenditure and the prevalence of MTLCs. Therefore, our findings should be viewed as preliminary and hypothesis-generating. Further, our regression model does not consider other factors that may influence social care expenditure (e.g., intensity of social care service use or its type, length of stay in social care settings, or number of visits or interventions). In addition, we used only a single year of data. Future studies could helpfully explore the association between social care expenditure and the prevalence of MTLCs using longitudinal datasets. This would enable better scrutiny of any moves from home to care settings and possible hospital stays.

We used LA-level data as covariates and outcome in our model. As in other ecological studies, these data are prone to ecological fallacy and therefore do not reflect individual-level social care expenditure. It is probable that some of our study population paid for social care privately, in full or in part, which is not captured in aggregate-level LA social care expenditure. Where feasible, future studies should include the proportion of people eligible for social care funding in each LA, as well as those paying for social care privately and those who have combinations of funding that could range from self or family funding to NHS entitlements and disability benefits, both means-tested and non-means tested. The role of family care inputs might also be explored if data allowed for this. Our study relied on routinely collected death registry data, which is not collected for research purposes and is therefore prone to bias.^48,49^ For example, death registry data has been prone to inaccuracies in terms of recording of the conditions and location of death.^50^ In addition, we used contributory causes of death as a proxy for the proportion of people with MLTC in the population due to a lack of incidence data. Future studies should use appropriate incidence data of MTLCs to examine the association with social care expenditure. Finally, our study concentrates on the minority for older people with MLTCs, understanding factors associated with older people living well would also be worth illuminating.

## Conclusions

The findings from this area-level analysis show that LA social care expenditure is linked to the prevalence of MTLCs. A higher prevalence of MLTCs among people aged ≥ 65 years increases social care expenditure, even after adjusting for LA deprivation scores, *per capita* income, median LA age, gender, and regional variation. This finding should be interpreted with caution as our study design is observational, and our model does not include several factors that may influence social care expenditures. As a result, we can only interpret our findings as hypothesis generating. It is important for future studies to further explore the mechanisms or link between social care expenditure and prevalence of MTLCs by using appropriate individual level datasets but of course it is even more important to prevent or reduce the risk of MTLCs and their harm. Cardiovascular disease was the most prevalent condition, co-existing with conditions such as diabetes, dementia, CKD, and respiratory diseases. This result suggests that management and treatment of these conditions must transcend traditional boundaries of care or specialties that focus on a single condition. Our data also showed that certain conditions are concentrated in poorer or more deprived areas. Better care integration and coordination, including policy reforms or initiatives targeting patients with these MLTC conditions living in deprived areas, could reduce inequalities. In addition, hospitals were the main place of death for older people with or without MLTCs. The high proportion of hospital deaths is perhaps due to a lack of access to community-based palliative care and/or social care services. Future research is warranted to validate these findings using appropriate longitudinal datasets.

## Supplemental Material

Supplemental Material - The association between social care expenditure and multiple-long term conditions: A population-based area-level analysis Click here for additional data file. Supplemental Material for The association between social care expenditure and multiple-long term conditions: A population-based area-level analysis by Emeka Chukwusa, Paulino Font-Gilabert, Jill Manthorpe and Andrew Healey in Journal of Multimorbidity and Comorbidity

## References

[1] Marengoni A. , et al., Aging with multimorbidity: a systematic review of the literature*.* Ageing Res Rev, 2011. 10(4): p. 430-439. 21402176 10.1016/j.arr.2011.03.003

[2] Nguyen H. , et al., Prevalence of multimorbidity in community settings: A systematic review and meta-analysis of observational studies*.* J Comorb, 2019. 9: p. 2235042x19870934. 10.1177/2235042X19870934 PMC6710708 31489279

[3] Kingston A. , et al., Projections of multi-morbidity in the older population in England to 2035: estimates from the Population Ageing and Care Simulation (PACSim) model*.* Age Ageing, 2018. 47(3): p. 374-380. 29370339 10.1093/ageing/afx201 PMC5920286

[4] Soley-Bori M. , et al., Impact of multimorbidity on healthcare costs and utilisation: a systematic review of the UK literature*.* Br J Gen Pract, 2021. 71(702): p. e39-e46. 33257463 10.3399/bjgp20X713897 PMC7716874

[5] Leniz J. , et al., Exploring costs, cost components, and associated factors among people with dementia approaching the end of life: A systematic review*.* Alzheimers Dement (N Y), 2021. 7(1): p. e12198. 34541291 10.1002/trc2.12198 PMC8438684

[6] Lehnert T. , et al., Review: health care utilization and costs of elderly persons with multiple chronic conditions*.* Med Care Res Rev, 2011. 68(4): p. 387-420. 21813576 10.1177/1077558711399580

[7] Hajat C. Siegal Y. Adler-Waxman A. , Clustering and Healthcare Costs With Multiple Chronic Conditions in a US Study*.* Front Public Health, 2020. 8: p. 607528. 33553094 10.3389/fpubh.2020.607528 PMC7859629

[8] Doos L. , et al., Mosaic segmentation, COPD and CHF multimorbidity and hospital admission costs: a clinical linkage study*.* J Public Health (Oxf), 2014. 36(2): p. 317-324. 23903003 10.1093/pubmed/fdt070

[9] Stokes J. , et al., Cuts to local government spending, multimorbidity and health-related quality of life: A longitudinal ecological study in England*.* Lancet Reg Health Eur, 2022. 19: p. 100436. 36039277 10.1016/j.lanepe.2022.100436 PMC9417904

[10] Public Health England , The health and social care costs of a selection of health conditions and multi-morbidities, Public Health England, 2020.

[11] Tran P.B. , et al., Costs of multimorbidity: a systematic review and meta-analyses*.* BMC Medicine, 2022. 20(1): p. 234. 35850686 10.1186/s12916-022-02427-9 PMC9295506

[12] The King's Fund . Social care in a nutshell - Eligibility for publicly funded social care. 2023 [cited 31/07/23]; Available from: https://www.kingsfund.org.uk/projects/nhs-in-a-nutshell/social-care-nutshell.

[13] Simon Bottery Deborah Fenney , Social care 360. 2019 London, The King's Fund.

[14] Hood R. , et al., Patterns of Demand and Provision in English Adult Social Care Services*.* The British Journal of Social Work, 2022. 52(7): p. 3858-3880.

[15] Benchimol E.I. , et al., The REporting of studies Conducted using Observational Routinely-collected health Data (RECORD) statement*.* Zeitschrift fur Evidenz, Fortbildung und Qualitat im Gesundheitswesen, 2016. 115: p. 33-48. 27837958 10.1016/j.zefq.2016.07.010 PMC5330542

[16] Divo M.J. Martinez C.H. Mannino D.M. , Ageing and the epidemiology of multimorbidity*.* Eur Respir J, 2014. 44(4): p. 1055-1068. 25142482 10.1183/09031936.00059814 PMC4918092

[17] Beattie J.M. , et al., Implementation of the Mental Capacity Act: a national observational study comparing resultant trends in place of death for older heart failure decedents with or without comorbid dementia*.* BMC Med, 2022. 20(1): p. 30. 35057803 10.1186/s12916-021-02210-2 PMC9901524

[18] Henson L.A. , et al., Lung cancer deaths (England 2001-2017)-comorbidities: a national population-based analysis*.* BMJ Support Palliat Care, 2021. 10.1136/bmjspcare-2021-003107 34489325

[19] NHS Digital . Adult Social Care Activity and Finance Report, England - 2018-19. 2019 August 2022];Available from: https://digital.nhs.uk/data-and-information/publications/statistical/adult-social-care-activity-and-finance-report/2018-19.

[20] Dodsworth E.O.C. , How much social care does each country fund?, in Adult social care in the four countries of the UK. Explainer series, in Nuffield Trust. 2023.

[21] NHS Digital . Adult Social Care Activity and Finance Report, *England - 2018-19 [PAS]*. 2019 [cited 2022 30/09/2022]; Available from: https://digital.nhs.uk/data-and-information/publications/statistical/adult-social-care-activity-and-finance-report/2018-19.

[22] ONS. Population Estimates for UK, England and Wales, Scotland and Northern Ireland: mid-2018, *using pre-April 2019 local authority district geography* 2019 August 2022]; Available from: https://www.ons.gov.uk/peoplepopulationandcommunity/populationandmigration/populationestimates/datasets/populationestimatesforukenglandandwalesscotlandandnorthernireland.

[23] DLUHC, National Statistics - Local authority revenue expenditure and financing England: 2021 to 2022 final outturn (Revised). 2023.

[24] Hazra N.C. Rudisill C. Gulliford M.C. , Determinants of health care costs in the senior elderly: age, comorbidity, impairment, or proximity to death? The European Journal of Health Economics, 2018. 19(6): p. 831-842. 28856487 10.1007/s10198-017-0926-2 PMC6008359

[25] Team, R.C. , R: A Language and Environment for Statistical Computing*.* 2020.

[26] McLean G. , et al., The influence of socioeconomic deprivation on multimorbidity at different ages: a cross-sectional study*.* British Journal of General Practice, 2014. 64(624): p. e440. 10.3399/bjgp14X680545 PMC4073730 24982497

[27] Mercer S.W. , et al., Multimorbidity and Socioeconomic Deprivation in Primary Care Consultations*.* Ann Fam Med, 2018. 16(2): p. 127-131. 29531103 10.1370/afm.2202 PMC5847350

[28] Head A. , et al., Inequalities in incident and prevalent multimorbidity in England, 2004-2013;19: a population-based, descriptive study*.* The Lancet Healthy Longevity, 2021. 2(8): p. e489-e497. 36097998 10.1016/S2666-7568(21)00146-X

[29] Barnett K. , et al., Epidemiology of multimorbidity and implications for health care, research, and medical education: a cross-sectional study*.* Lancet, 2012. 380(9836): p. 37-43. 22579043 10.1016/S0140-6736(12)60240-2

[30] Katikireddi S.V. , et al., The contribution of risk factors to socioeconomic inequalities in multimorbidity across the lifecourse: a longitudinal analysis of the Twenty-07 cohort*.* BMC Med, 2017. 15(1): p. 152. 28835246 10.1186/s12916-017-0913-6 PMC5569487

[31] Grundy E.M. Stuchbury R. , Multimorbidity as assessed by reporting of multiple causes of death: variations by period, sociodemographic characteristics and place of death among older decedents in England and Wales, 2001–2017*.* Journal of Epidemiology and Community Health, 2022. 76(8): p. 699. 35654580 10.1136/jech-2021-217846 PMC9279827

[32] Akugizibwe R. , et al., Multimorbidity Patterns and Unplanned Hospitalisation in a Cohort of Older Adults*.* J Clin Med, 2020. 9(12), 233. 33321977 10.3390/jcm9124001 PMC7764652

[33] Seow H. , et al., Impact of community based, specialist palliative care teams on hospitalisations and emergency department visits late in life and hospital deaths: a pooled analysis*.* BMJ, 2014. 348: p. g3496. 24906901 10.1136/bmj.g3496 PMC4048125

[34] Chukwusa E. , et al., Urban and rural differences in geographical accessibility to inpatient palliative and end-of-life (PEoLC) facilities and place of death: a national population-based study in England, UK*.* Int J Health Geogr, 2019. 18(1): p. 8. 31060555 10.1186/s12942-019-0172-1 PMC6503436

[35] Chukwusa E. , et al., Regional variations in geographic access to inpatient hospices and Place of death: A Population-based study in England, UK*.* PLoS One, 2020. 15(4): p. e0231666. 32302344 10.1371/journal.pone.0231666 PMC7164606

[36] Gonzalez-Gonzalez A.I. , et al., End-of-Life Care Preferences of Older Patients with Multimorbidity: A Mixed Methods Systematic Review*.* J Clin Med, 2020. 10(1). 10.3390/jcm10010091 PMC7795676 33383951

[37] Kasteridis P. , et al. The importance of multimorbidity in explaining utilisation and costs across health and social care settings: evidence from South Somersets Symphony Project. 2014.

[38] Nikolova S. , et al., Social care costs for community-dwelling older people living with frailty*.* Health Soc Care Community, 2022. 30(3): p. e804-e811. 34080751 10.1111/hsc.13450

[39] Local Government Association , Explaining Variation in Spending – Adults’ Services for Older People. 2020.

[40] Crawford R. Phillips D. Local government spending: where is the axe falling? In: Emmerson C Johnson P Miller H (eds) The IFS Green Budget: February 2012. London: The Institute for Fiscal Studies, 124–141. 2012.

[41] Foster D. Harker R. , Adult social care funding(England). 2023 https://commonslibrary.parliament.uk/research-briefings/cbp-7903/.

[42] Watkins J. , et al., Effects of health and social care spending constraints on mortality in England: a time trend analysis*.* BMJ Open, 2017. 7(11): p. e017722. 10.1136/bmjopen-2017-017722 PMC5719267 29141897

[43] Melin Emilsson U. Strid A.-L. Söderberg M. , Lack of Coordination between Health Care and Social Care in Multi-Professional Teamwork - the Obstacle for Coherent Care of Older People Suffering from Multi-Morbidity*.* Journal of Population Ageing, 2022. 15(2): p. 319-335.

[44] Mercer S.W. , et al., The CARE Plus study – a whole-system intervention to improve quality of life of primary care patients with multimorbidity in areas of high socioeconomic deprivation: exploratory cluster randomised controlled trial and cost-utility analysis*.* BMC Medicine, 2016. 14(1): p. 88. 27328975 10.1186/s12916-016-0634-2 PMC4916534

[45] Zhou Y. , et al., Interventions and management on multimorbidity: An overview of systematic reviews*.* Ageing Res Rev, 2023. 87: p. 101901. 36905961 10.1016/j.arr.2023.101901

[46] Thomas P. , The structure of the NHS in England. 2020. https://allcatsrgrey.org.uk/wp/download/health_services/CBP-7206.pdf.

[47] Wang H. , et al., Estimating excess mortality due to the COVID-19 pandemic: a systematic analysis of COVID-19-related mortality, 2020-;21*.* The Lancet, 2022. 399(10334): p. 1513-1536. 10.1016/S0140-6736(21)02796-3 PMC8912932 35279232

[48] Rezel-Potts E. Gulliford M. , Electronic Health Records and Antimicrobial Stewardship Research: a Narrative Review*.* Curr Epidemiol Rep, 2022: p. 1-10. 10.1007/s40471-021-00278-1 PMC9303046 35891969

[49] Davies J.M. , et al., Using routine data to improve palliative and end of life care*.* BMJ Support Palliat Care, 2016. 6(3): p. 257-262. 10.1136/bmjspcare-2015-000994 PMC5013160 26928173

[50] Cohen J. , et al., Using death certificate data to study place of death in 9 European countries: opportunities and weaknesses*.* BMC Public Health, 2007. 7(1): p. 283. 17922894 10.1186/1471-2458-7-283 PMC2099436

