# Supplementary material for: The association between social care expenditure and multiple-long term conditions: A population-based area-level analysis
Source: J Multimorb Comorb. 2023 Oct 27;13:26335565231208994. doi: 10.1177/26335565231208994 (PMC10612455; doi:10.1177/26335565231208994)
Supplement: Supplemental Material - The association between social care expenditure and multiple-long term conditions: A population-based area-level analysis [file sj-pdf-1-cob-10.1177_26335565231208994.pdf]

Table S1 Combinations of ≥ 2 MTLCs

Table S2: Association between MTLCs and LA social care expenditure. Alternative order

S\N	MTLCs	Prevalence (%)
1	CVDs-Diabetes	15.42
2	CVDs-Dementia	15.34
3	CVDs-Respiratory	14.53
4	CVDs-CKD	12.77
5	CVDs-Diabetes-CKD	5.59
6	Dementia-Respiratory	3.34
7	Dementia-Diabetes	2.93
8	CVDs-Dementia-CKD	2.91
9	CVDs-Diabetes-Respiratory	2.63
10	Dementia-CKD	2.53
11	Diabetes-CKD	2.48
12	CVDs-Dementia-Diabetes	2.39
13	CVDs-CKD-Respiratory	2.23
14	Diabetes-Respiratory	1.66
15	CVDs-Dementia-Respiratory	1.57
16	CVDs-Diabetes-Stroke	1.35
17	CVDs-Dementia-Stroke	1.29
18	CKD-Respiratory	1.28
19	CVDs-Dementia-Diabetes-CKD	0.80
20	CVDs-Diabetes-CKD-Respiratory	0.79
21	CVDs-CKD-Stroke	0.76
22	CVDs-Respiratory-Stroke	0.69
23	Dementia-Diabetes-CKD	0.64
24	CVDs-Diabetes-CKD-Stroke	0.39
25	Dementia-Diabetes-Stroke	0.32
26	Diabetes-CKD-Respiratory	0.30
27	CVDs-Dementia-Diabetes-Stroke	0.28
28	CVDs-Dementia-CKD-Respiratory	0.27
29	Dementia-Diabetes-Respiratory	0.26
30	CVDs-Dementia-Diabetes-Respiratory	0.25
31	CVDs-Dementia-CKD-Stroke	0.24
32	Dementia-CKD-Respiratory	0.22
33	Dementia-CKD-Stroke	0.18
34	Dementia-Respiratory-Stroke	0.18
35	CVDs-Diabetes-Respiratory-Stroke	0.16
36	Diabetes-CKD-Stroke	0.15
37	CVDs-Dementia-Respiratory-Stroke	0.14
38	Diabetes-Respiratory-Stroke	0.12
39	CVDs-CKD-Respiratory-Stroke	0.11
40	CVDs-Dementia-Diabetes-CKDStroke	0.09
41	CVDs-Dementia-Diabetes-CKD-Respiratory	0.07
42	Dementia-Diabetes-CKD-Stroke	0.06
43	CKD-Respiratory-Stroke	0.06
44	Dementia-Diabetes-CKD-Respiratory	0.05
45	CVDs-Diabetes-CKD-Respiratory-Stroke	0.04
46	CVDs-Dementia-Diabetes-Respiratory-Stroke	0.03
47	CVDs-Dementia-CKD-Respiratory-Stroke	0.02
48	Dementia-Diabetes-Respiratory-Stroke	0.02
49	Diabetes-CKD-Respiratory-Stroke	0.01
50	Dementia-CKD-Respiratory-Stroke	0.01
51	CVDs-Dementia-Diabetes-CKD-Respiratory-Stroke	0.01
52	Dementia-Diabetes-CKD-Respiratory-Stroke	0.00

	social care expenditure		
VARIABLES	(1)	(2)	(3)
MLTC pp		8.67**	8.13**
		(1.00 - 16.33)	(0.24 - 16.02)
LA income pc	0.03*	0.03*	0.01
	(-0.00 - 0.05)	(-0.00 - 0.06)	(-0.02 - 0.05)
IMD - Average score	16.92***	15.36***	15.86***
	(11.96 - 21.88)	(10.34 - 20.38)	(10.82 - 20.90)
Share female (pp)			-36.00
			(-119.95 - 47.94)
Median age (65+)			-4.78
			(-79.76 - 70.20)
Constant	456.02***	319.76***	2,634.47
	(298.26 - 613.78)	(128.55 - 510.96)	(-1,805.59 - 7,074.53)
Observations	148	148	148
R-squared	0.37	0.39	0.40
Region FE	YES	YES	YES

Robust ci in parentheses
*** p<0.01, ** p<0.05, * p<0.1

*Results from OLS regressions. MLTC pp is the prevalence of multiple long-term conditions expressed in percentage points. LA income pc is the local authority income per capita. Deprivation score is the index of multiple deprivation, share female (pp) is the proportion of female population. A positive coefficient reflects a positive association between variables, while a negative coefficient reflects a negative association. Robust confidence Interval in parentheses*** p<0.01, ** p<0.05, * p<0.1*

Table S3: Correlations of variables

	SC expend 65+ (pc)	MLTC 65+	LA Income	Deprivation Score	Share 65+	Median age 65+
**SC expend 65+ (pc)**	1					
**MLTC 65+**	0.2746	1				
**LA Income**	0.2018	0.0008	1			
**Deprivation Score**	0.3781	0.1677	-0.0411	1		
**Share 65+**	0.0059	0.1227	-0.4584	0.0735	1	
**Median age 65+**	-0.1814	-0.0218	-0.1069	-0.2706	0.2185	1

Table S4: Breakdown of Local Authorities within Regions

Region	Local Authorities
**East Midlands**	Derby
	Derbyshire
	Leicester
	Leicestershire
	Lincolnshire
	Northamptonshire
	Nottingham
	Nottinghamshire
	Rutland
**East of England**	Bedford
	Cambridgeshire
	Central Bedfordshire
	Essex
	Hertfordshire
	Luton
	Norfolk
	Peterborough
	Southend-on-Sea
	Suffolk
	Thurrock
**London**	Barking and Dagenham
	Barnet
	Bexley
	Brent
	Bromley
	Camden
	City of London
	Croydon
	Ealing
	Enfield
	Greenwich
	Hackney
	Hammersmith and Fulham
	Haringey
	Harrow
	Havering
	Hillingdon
	Hounslow
	Islington
	Kensington and Chelsea
	Kingston upon Thames
	Lambeth
	Lewisham
	Merton
	Newham
	Redbridge

	Richmond upon Thames
	Southwark
	Sutton
	Tower Hamlets
	Waltham Forest
	Wandsworth
	Westminster
**North East**	County Durham
	Darlington
	Gateshead
	Hartlepool
	Middlesbrough
	Newcastle upon Tyne
	North Tyneside
	Northumberland
	Redcar and Cleveland
	South Tyneside
	Stockton-on-Tees
	Sunderland
**North West**	Blackburn with Darwen
	Blackpool
	Bolton
	Bury
	Cheshire East
	Cheshire West and Chester
	Cumbria
	Halton
	Knowsley
	Lancashire
	Liverpool
	Manchester
	Oldham
	Rochdale
	Salford
	Sefton
	St. Helens
	Stockport
	Tameside
	Trafford
	Warrington
	Wigan
	Wirral
**South East**	Bracknell Forest
	Brighton and Hove
	Buckinghamshire
	East Sussex
	Hampshire
	Isle of Wight

	Kent
	Medway
	Milton Keynes
	Oxfordshire
	Portsmouth
	Reading
	Slough
	Southampton
	Surrey
	West Berkshire
	West Sussex
	Windsor and Maidenhead
	Wokingham
**South West**	Bath and North East Somerset
	Bournemouth
	Bristol City of
	Cornwall
	Devon
	Dorset
	Gloucestershire
	Isles of Scilly
	North Somerset
	Plymouth
	Poole
	Somerset
	South Gloucestershire
	Swindon
	Torbay
	Wiltshire
**West Midlands**	Birmingham
	Coventry
	Dudley
	Herefordshire County of
	Sandwell
	Shropshire
	Solihull
	Staffordshire
	Stoke-on-Trent
	Telford and Wrekin
	Walsall
	Warwickshire
	Wolverhampton
	Worcestershire
**Yorkshire and The Humber**	Barnsley
	Bradford
	Calderdale
	Doncaster

	East Riding of Yorkshire
	Kingston upon Hull City of
	Kirklees
	Leeds
	North East Lincolnshire
	North Lincolnshire
	North Yorkshire
	Rotherham
	Sheffield
	Wakefield
	York
